# Insomnia subtypes characterised by objective sleep duration and NREM spectral power and the effect of acute sleep restriction: an exploratory analysis

**DOI:** 10.1038/s41598-021-03564-6

**Published:** 2021-12-21

**Authors:** Chien-Hui Kao, Angela L. D’Rozario, Nicole Lovato, Rick Wassing, Delwyn Bartlett, Negar Memarian, Paola Espinel, Jong-Won Kim, Ronald R. Grunstein, Christopher J. Gordon

**Affiliations:** 1 CIRUS Centre for Sleep and Chronobiology, Woolcock Institute of Medical Research, Sydney, Australia; 2 School of Psychology, Faculty of Science, The University of Sydney, Sydney, NSW Australia; 3 Adelaide Institute for Sleep Health, College of Medicine and Public Health, Flinders University, Bedford Park, SA Australia; 4 Faculty of Medicine and Health, The University Sydney, Camperdown, Sydney, NSW 2050 Australia; 5 British Columba Children’s Hospital Research Institute, Vancouver, Canada; 6 Department of Healthcare IT, Inje University, Inje, South Korea; 7 Sleep and Severe Mental Illness Clinic, CPC-RPA Clinic, Royal Prince Alfred Hospital, Sydney, NSW Australia

**Keywords:** Neurophysiology, Sleep disorders, Electroencephalography - EEG

## Abstract

Insomnia disorder (ID) is a heterogeneous disorder with proposed subtypes based on objective sleep duration. We speculated that insomnia subtyping with additional power spectral analysis and measurement of response to acute sleep restriction may be informative in overall assessment of ID. To explore alternative classifications of ID subtypes, insomnia patients (n = 99) underwent two consecutive overnight sleep studies: (i) habitual sleep opportunity (polysomnography, PSG) and, (ii) two hours less sleep opportunity (electroencephalography, EEG), with the first night compared to healthy controls (n = 25). ID subtypes were derived from data-driven classification of PSG, EEG spectral power and interhemispheric EEG asymmetry index. Three insomnia subtypes with different sleep duration and NREM spectral power were identified. One subtype (n = 26) had shorter sleep duration and lower NREM delta power than healthy controls (short-sleep delta-deficient; SSDD), the second subtype (n = 51) had normal sleep duration but lower NREM delta power than healthy controls (normal-sleep delta-deficient; NSDD) and a third subtype showed (n = 22) no difference in sleep duration or delta power from healthy controls (normal neurophysiological sleep; NNS). Acute sleep restriction improved multiple objective sleep measures across all insomnia subtypes including increased delta power in SSDD and NSDD, and improvements in subjective sleep quality for SSDD (*p* = 0.03), with a trend observed for NSDD (*p* = 0.057). These exploratory results suggest evidence of novel neurophysiological insomnia subtypes that may inform sleep state misperception in ID and with further research, may provide pathways for personalised care.

## Introduction

Patients with insomnia disorder (ID) universally report sleep disturbances such as problems initiating or maintaining sleep, or dissatisfaction with sleep quality. According to a recent survey, the prevalence of insomnia in the general population ranges from 5 to 33%, with 12% of insomnia diagnosed according to DSM-5 criteria^42^ in Australia. However, these subjective findings are often discordant with objectively-measured sleep using polysomnography (PSG)^5,12,43^. Research has shown that PSG-measured sleep metrics can be remarkably similar between insomnia patients and healthy-sleeping controls^7^ and are often unable to identify the subjective complaints associated with insomnia^44^. This lack of subjective–objective alignment in insomnia patients has diagnostic implications, with PSG studies not recommended as they do not improve clinical diagnosis^44^. However, analysis of the sleep electroencephalography (EEG) spectral power may provide greater insights into the subjective sleep disturbance which is the central feature of insomnia. Insomnia patients have been shown to have increased non-rapid eye movement (NREM) high-frequency EEG spectral power (beta-range) compared to controls^35,40,49^, and decreased delta or slow wave activity (SWA) during NREM sleep^14,29^, although, these findings have not been replicated by others^4,25^. More recent evidence showed that insomnia patients have lower initial NREM low-frequency power, and that the normal dissipation of low-frequency power across the night is compromised^31^. Combined, these results suggest that EEG spectral power during sleep, in contrast to standard polysomnography, may provide a better biomarker distinguishing insomnia subtypes^57^, relate to subjective insomnia symptoms^30^ and provide more informative with regard to personalised care in ID.

Whilst these studies have shown differences in global EEG spectral power between insomnia and controls, it is known that regional brain areas are not in the same vigilance state at any given point in time^47^. Interhemispheric EEG asymmetry results when SWA dominates in one hemisphere, whilst the other shows fast frequency EEG activity^26,48^. Interhemispheric asymmetrical sleep has been shown to occur in healthy individuals when exposed to sleeping in an unfamiliar environment, with a reduction in SWA in the left hemispheric compared to the right^52^. This may explain why on the first night in an unfamiliar environment, healthy sleepers can experience a ‘first night effect’ where sleep patterns are altered compared to usual sleep. However, studies are inconsistent in their findings and some have revealed no differences in sleep pattern between insomnia patients and good sleepers^9,19,39^. Moreover, certain insomnia patients experience ‘reverse first-night effect, with a reported overestimation of sleep^17^.

In insomnia patients, intra-individual variability of interhemispheric asymmetry has been observed^27^ with individuals switching dominance between hemispheres across a night’s sleep. Evidence shows that in addition to interhemispheric EEG asymmetry, insomnia had greater fronto-parietal asymmetry in the left hemisphere, particularly in slow frequency bands (delta and theta) during the REM sleep, when compared to the healthy controls^41^. Furthermore, asymmetry was also observed in several insomnia subtypes (paradoxical insomnia and psychophysiological insomnia)^50,51^. It was shown that paradoxical insomnia had higher asymmetry in the frontal region of the right hemisphere compared to the left, whereas psychophysiological insomnia had greater asymmetry in the parietal area. Accordingly, interhemispheric EEG asymmetry in insomnia may contribute to cortical hyperarousal and explain sleep-state misperception.

The lack of subjective–objective alignment in insomnia may also be related to the considerable heterogeneity observed in insomnia populations. We have shown differences in EEG spectral power at sleep onset in insomnia subtypes^36^ with a short-sleep duration insomnia subtype exhibiting reduced alpha, beta and delta spectral power before sleep onset compared to a normal-sleep duration insomnia subtype. Insomnia patients with subjective–objective discrepancies exhibit decreased delta and increased sigma, alpha, and beta power during NREM compared to those with concordant subjective–objective sleep duration^29^. However, there are a paucity of studies exploring PSG and quantitative EEG-derived (qEEG) insomnia subtypes that may explain the subjective symptomology reported by insomnia patients.

Given a lack of research on characterising neurophysiological features of insomnia, there is a need to better understand how insomnia subtypes are different and respond to sleep challenges. Therefore, this exploratory study aims to categorise neurophysiological subtypes of insomnia using PSG, spectral power, and interhemispheric asymmetry, and compare the objective and subjective matrices between the insomnia subtypes and healthy controls during habitual sleep. We also explore how insomnia subtypes respond to acute sleep restriction night (2-h reduced bedtime). Overall, this exploratory study aims to expand our understanding of insomnia neurophysiology and help explain objective-subjective discrepancies.

## Results

There were 99 insomnia and 25 healthy control participants with complete PSG and quantitative EEG data from the habitual sleep opportunity (night 1) study. 84 insomnia participants completed the second consecutive acute sleep restriction study consisting of 2-h reduced total bedtime.

### Insomnia subtype description

Insomnia subtypes were constructed using clustering analysis based on the PSG, delta EEG spectral power, and interhemispheric asymmetry index (IAI) of insomnia data from the first sleep study (night 1) and compared with controls. Overall, the first subtype (n = 26) showed abnormalities in both sleep macro-architecture as well as EEG power-spectral measures and will be labelled “*short-sleep delta-deficient subtype (SSDD)*”. The second subtype (n = 51) showed no apparent changes to sleep macro-architecture but did show compromised EEG delta spectral power, labelled “*normal-sleep delta-deficient (NSDD) subtype*”. The third subtype (n = 22) showed no apparent changes in sleep macro-architecture or EEG power-spectral measures and exhibited similar sleep to healthy controls which has been termed “*normal neurophysiological sleep subtype (NNS)*”.

#### Demographics and clinic characteristics

The distribution of demographic and clinical characteristics of the overall insomnia group, insomnia subtypes and the controls are shown in Table 1. Using MANOVA model analysis, we found that the overall insomnia group was significantly older and had higher ISI, PSQI, sleepiness and DASS scores compared to controls. Additionally, SSDD were older compared to NNS (*p* = 0.02), but all other variables were similar between the insomnia subtypes (all *p* > 0.05).

**Table 1:** Demographics and clinic characteristics of insomnia subtypes and healthy controls.

	Total insomnia	Control	Insomnia subtypes			*p* values	Effect size
			SSDD	NSDD	NNS		
Sex (F)	n = 99 (69)	n = 25 (14)	n = 26 (18)	n = 51 (32)	n = 22 (19)	0.08	0.23
Age (y)	46.2 ± 1.5^d^	30.3 ± 1.7^abc^	52.9 ± 2.7^ cd^	44.9 ± 2.0^d^	41.4 ± 3.3^ad^	< 0.001	0.24
BMI (kg/m^2^)	25.4 ± 0.5	26.6 ± 1.0	25.7 ± 1.0	25.9 ± 0.7	23.9 ± 1.1	0.17	0.03
ISI	19.3 ± 0.8^d^	3.0 ± 0.6^abc^	20.8 ± 0.8^d^	19.8 ± 0.6^d^	18.8 ± 0.9^d^	< 0.00	0.50
PSQI	13.2 ± 0.3^d^	3.9 ± 0.5^abc^	14.5 ± 0.6^d^	12.5 ± 0.4^d^	13.2 ± 0.8^d^	< 0.001	0.55
DASS-D	9.1 ± 1.0^d^	1.6 ± 0.5^abc^	11.8 ± 2.5^d^	7.4 ± 1.2^d^	9.6 ± 0.5^d^	< 0.001	0.19
DASS-A	5.9 ± 0.7^d^	1.9 ± 0.6^abc^	7.4 ± 1.4^d^	4.6 ± 0.8^d^	7.2 ± 1.6^d^	0.003	0.13
DASS-S	13.8 ± 1.0^d^	2.9 ± 0.7^abc^	16.8 ± 2.3^d^	11.6 ± 1.1^d^	15.1 ± 2.2^d^	< 0.001	0.36
ESS	7.4 ± 0.6^d^	4.3 ± 0.6^b^	6.2 ± 1.0	8.2 ± 0.8^d^	6.9 ± 1.2	0.03	0.08
FSS	4.3 ± 0.2^d^	2.4 ± 0.2^abc^	4.5 ± 0.3^d^	4.1 ± 0.2^d^	4.5 ± 0.3^d^	< 0.001	0.26

Values are expressed as mean ± SEM.

*BMI* body mass index, *ISI* Insomnia severity index, *PSQI* Pittsburgh Sleep Quality Index, *DASS* Depression, Anxiety and Stress Scale (*D* depression, *A* Anxiety, *S *stress), *ESS* Epworth Sleepiness Scale, *FSS* Fatigue Severity Scale.

^a^Significantly different than subtype SSDD.

^b^Significantly different than subtype NSDD.

^c^Significantly different than subtype NNS.

^d^Significantly different than controls. Effect size based on Cramers phi and Kruskal–Wallis H test.

#### Sleep quality ratings and sleep-state misperception

**Figure 1:**
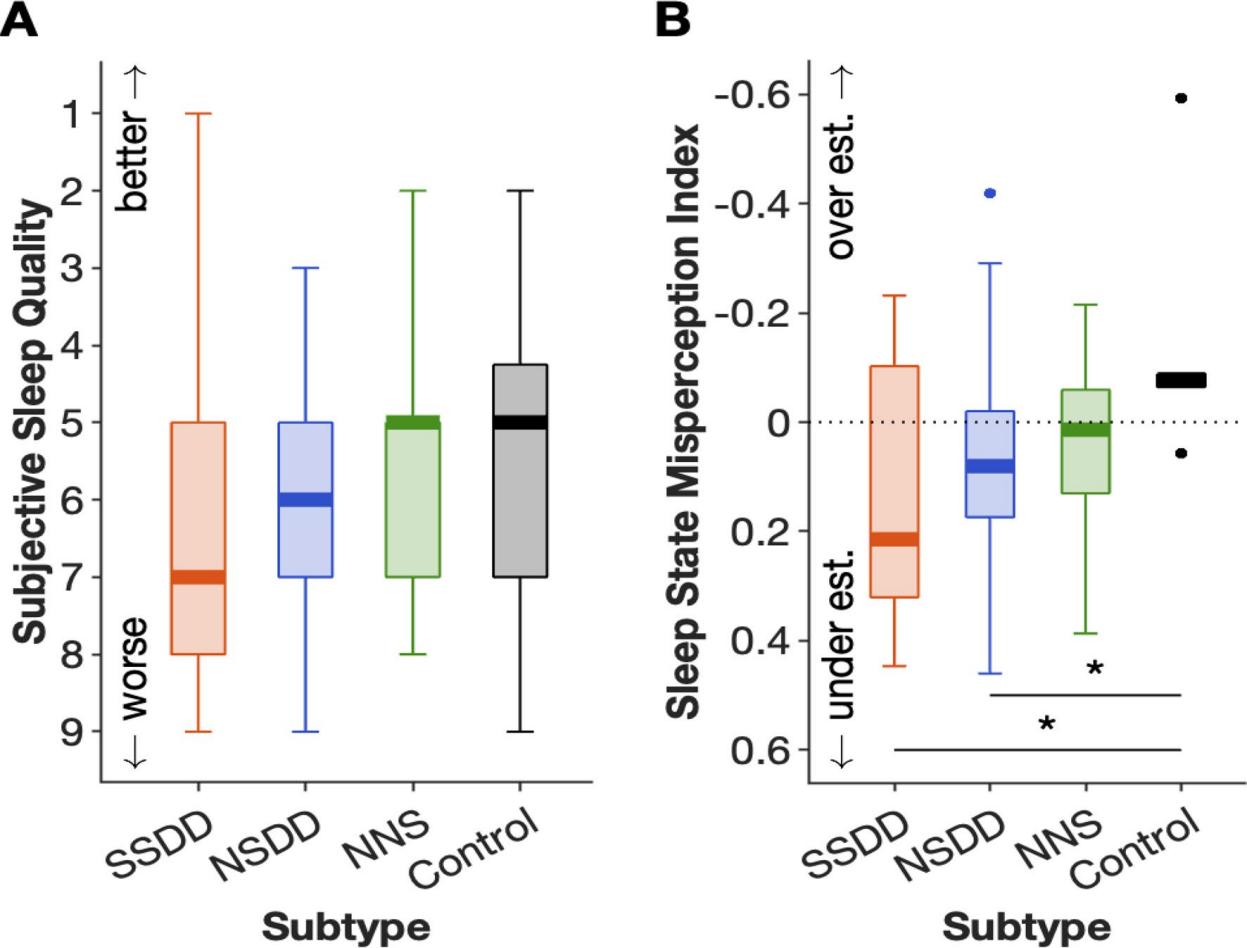
Short-sleeping and normal sleeping insomnia subtypes with delta-power deficiencies underestimate their total sleep time. Subjective sleep quality rating (**A**) and sleep state misperception (**B**) across insomnia subtypes and controls following the first night. The upper and lower whiskers indicate the value with Q3 + 1.5 × IQR and the value with Q1 − 1.5 × IQR, respectively. Black dots indicate outliers. *SSDD* short-sleep delta-deficient subtype, *NSDD* normal-sleep delta-deficient, *NNS* normal neurophysiological sleep subtype, *p < 0.05.

A MANCOVA analysis showed no significant differences in subjective sleep quality between the insomnia subtypes and controls (Fig. 1A). However, insomnia SSDD and NSDD, but not NNS, underestimated total sleep time (TST) compared to controls (Fig. 1B; sleep-state misperception index: 0.14, *p* = 0.02, and 0.05, *p* = 0.04, respectively).

#### Sleep macroarchitecture

**Figure 2:**
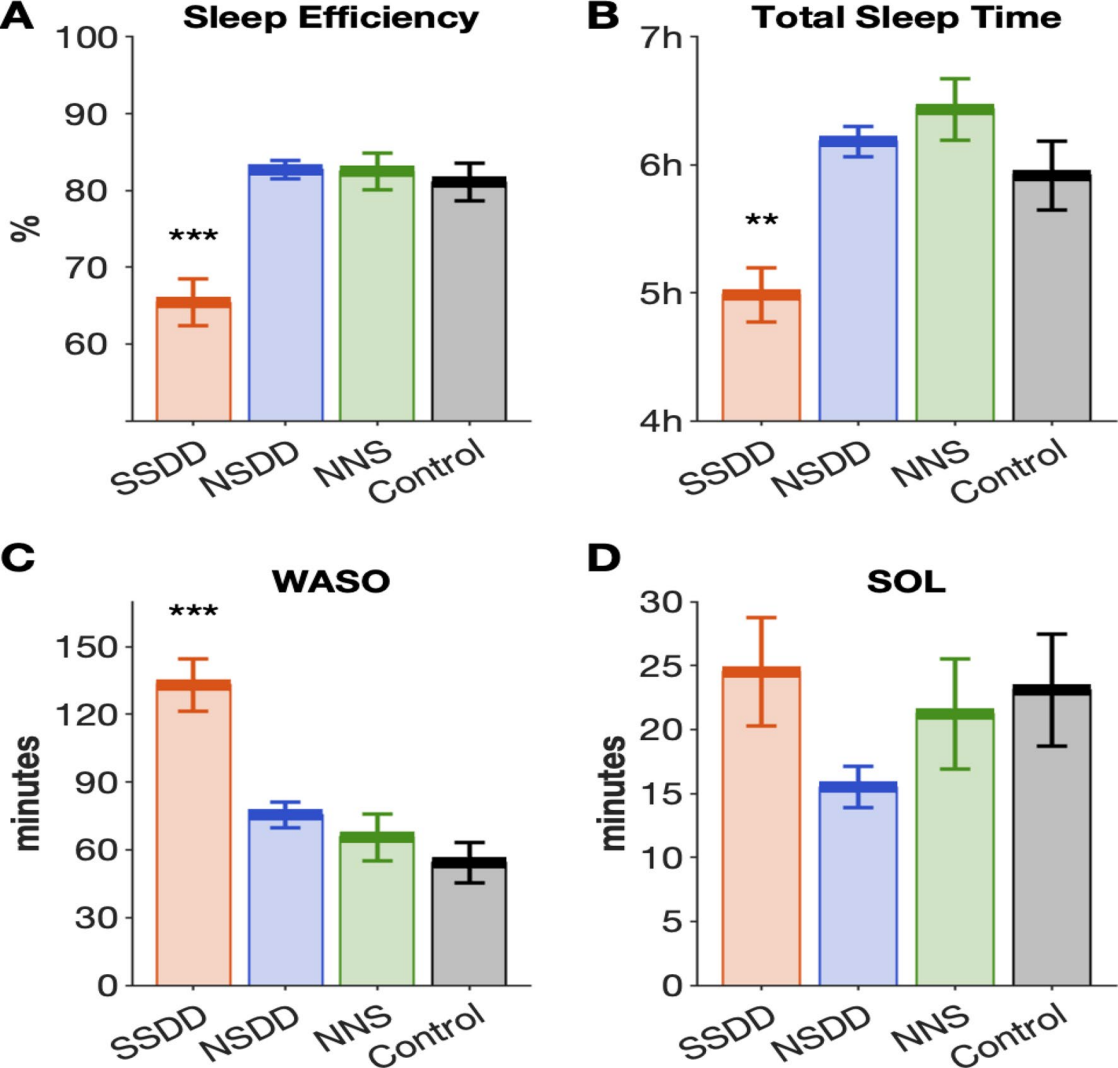
Only short-sleep delta-deficient insomnia subtype had worse macroarchitecture sleep. Polysomnographic sleep efficiency (**A**), total sleep time (**B**), wake after sleep onset (**C**), sleep onset latency (**D**) for insomnia subtypes and controls. Error bars indicate standard error of the mean, *SSDD* short-sleep delta-deficient subtype, *NSDD* normal-sleep delta-deficient, *NNS* normal neurophysiological sleep subtype, **p < 0.01, ***p < 0.001 denote the significant difference compared to the controls.

Only SSDD had significantly lower sleep efficiency (SE), shorter TST, and longer wake time after sleep onset (WASO) compared to controls (all *p* < 0.05). NSDD and NNS did not differ to the controls on SE, TST, WASO and sleep onset latency (SOL) (Fig. 2). Overall, there was no significant difference in NREM and rapid eye movement (REM) sleep between insomnia subtypes and controls, except SSDD had shorter REM duration, and more arousals than controls during TST and NREM but not during REM (Supplementary Table 1). These findings could not be explained by differences in time in bed (TIB) with no significant differences between the insomnia subtypes and controls: SSDD: 442.6 ± 58.5, NSDD: 450.7 ± 41.8, NNS: 467.4 ± 63.6, control: 434.3 ± 59.7 min (all *p* > 0.05).

#### Sleep EEG-power spectra

**Figure 3:**
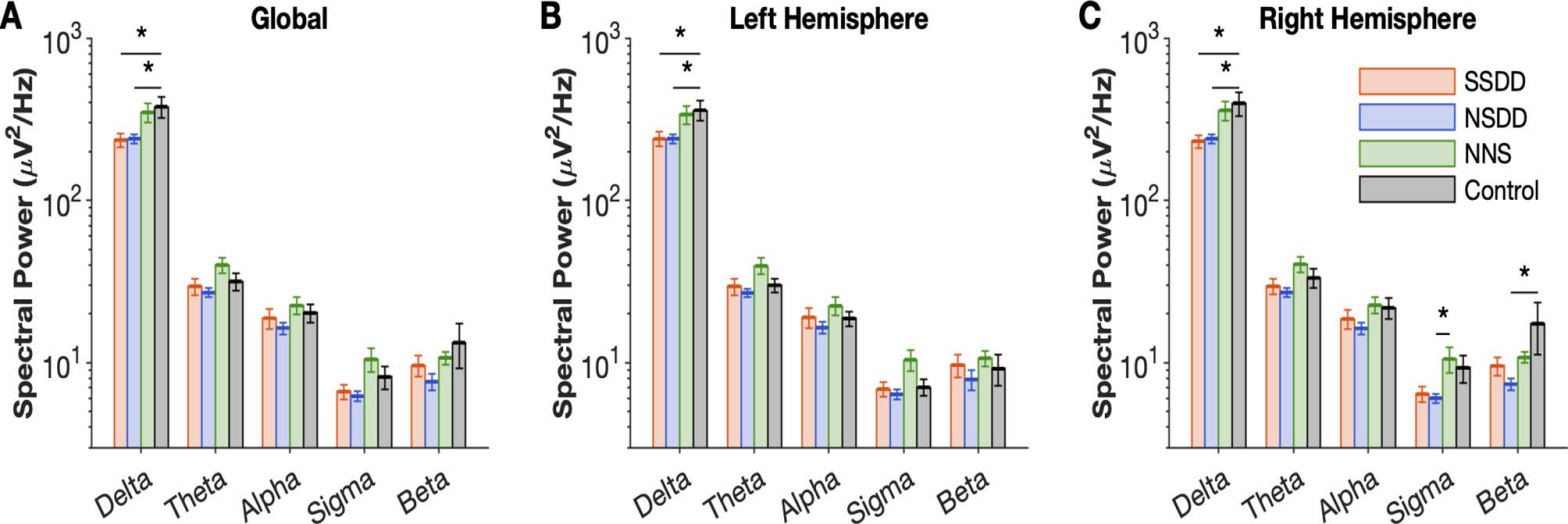
Two out of three insomnia subtypes have delta-power deficiencies during NREM sleep. Global EEG spectral power (**A**) across delta, theta, alpha, sigma and beta frequency bands during NREM sleep and in the right (**B**) and left hemispheres (**C**). Error bars indicate standard error of the mean, *SSDD* short-sleep delta-deficient subtype, *NSDD* normal-sleep delta-deficient, *NNS* normal neurophysiological sleep subtype, *p < 0.05 denotes the significant difference compared to controls.

In order to evaluate the difference in EEG spectral power between insomnia subtypes and controls, we used a MANCOVA model analysis to test the differences in 5 major EEG spectral frequency bands (*delta, theta, alpha, sigma, beta*). As compared to controls, SSDD and NSDD had significantly lower delta power in the in both the left and right hemispheres and globally (all *p* < 0.05, Fig. 3) during NREM sleep. Furthermore, these subtypes showed decreased delta power during REM sleep in both hemispheres (*p* < 0.01, Supplementary Table 2). *NSDD* also showed lower beta and sigma power in the right hemisphere during NREM sleep (*p* < 0.05, Fig. 3). Finally, *NNS* did not show any differences with controls across all spectral power frequencies in global or right/left hemispheres (all *p* > 0.05, Fig. 3).

#### Interhemispheric asymmetry index

We did not observe any differences between the insomnia subtypes and controls in interhemispheric asymmetry index (Supplementary Figure 1; all *p* > 0.05; SSDD: 0.007, NSDD: 0.0008, NNS: − 0.03, control: − 0.04).

Overall, the analysis of the three insomnia subtypes revealed that there were distinct differences in objective and subjective sleep variables between the subtypes, which are shown in Supplementary Table 3.

### Acute sleep restriction

**Figure 4:**
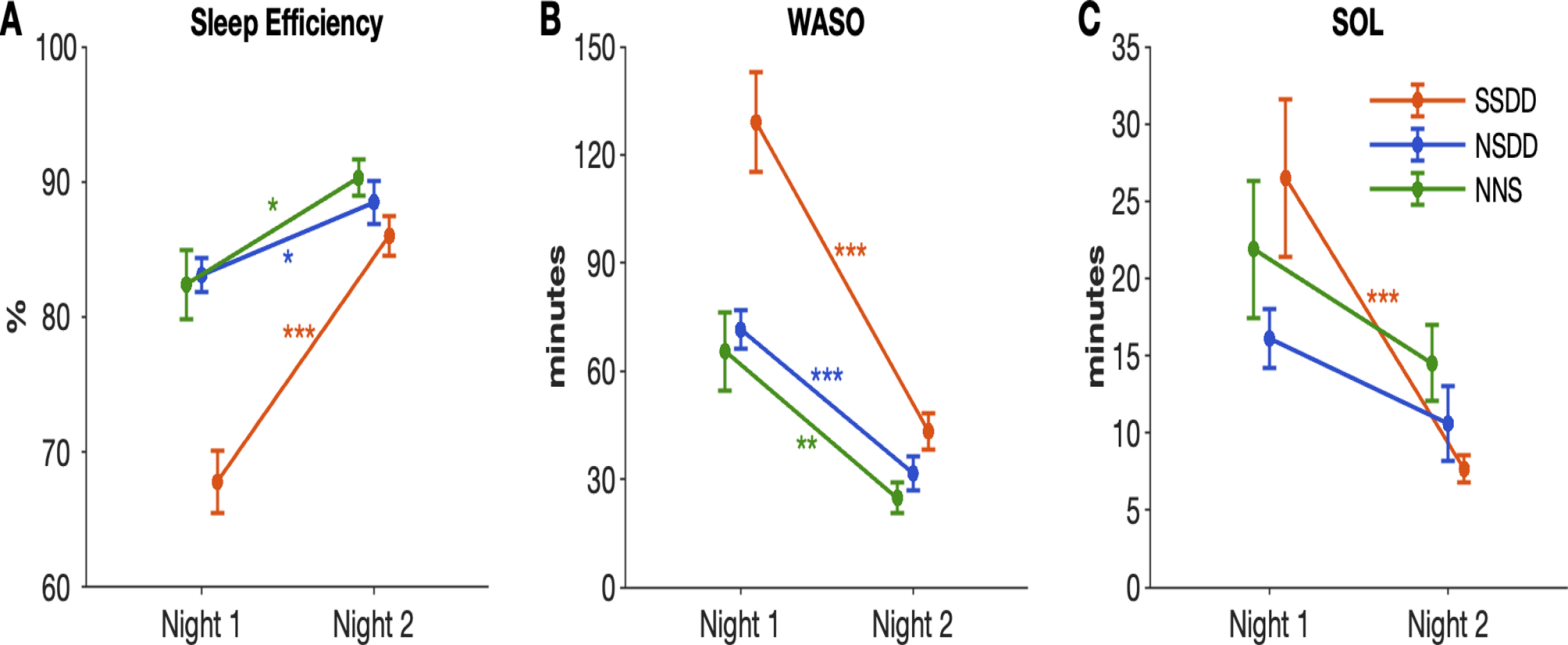
Improvement in sleep macroarchitecture with bedtime restriction. The change in sleep efficiency (**A**), wake after sleep onset (**B**), sleep onset latency (**C**) for the insomnia subtypes during habitual sleep (night 1) and acute sleep restriction (night 2). Error bars indicate standard error of the mean, *SSDD* short-sleep delta-deficient subtype, *NSDD* normal-sleep delta-deficient, *NNS* normal neurophysiological sleep subtype, *p < 0.05, **p < 0.01, ***p < 0.001.

On the consecutive second sleep study, acute sleep restriction was conducted by delaying habitual bedtime by two hours. This manipulation resulted in an increase in SE, SOL, and WASO in SSDD compared to the night one with full sleep opportunity (Fig. 4; all *p* < 0.001) by using a mixed-effect MANCOVA analysis. NSDD and NNS also had increased SE (*p* < 0.05) and reduced WASO (*p* < 0.001 and *p* < 0.01, respectively) compared to night one. All sleep architecture variables on night two are shown in Supplementary Table 4.

**Figure 5:**
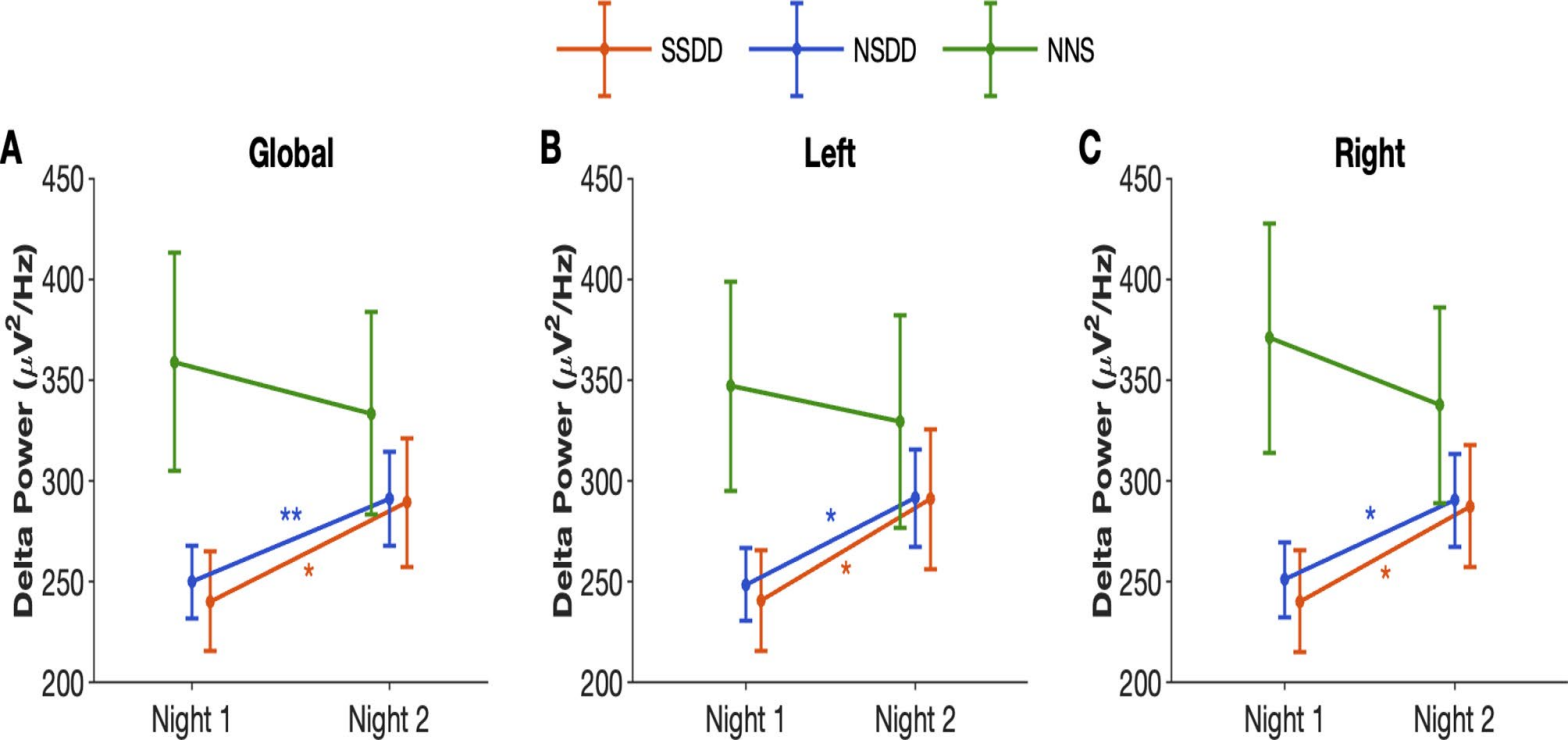
Both delta-deficient insomnia subtypes have stronger NREM delta-power with bedtime restriction. Global delta power during NREM sleep (**A**), in the right (**B**) and left hemisphere (**C**) between habitual sleep (night 1) and acute sleep restriction (night 2). Error bars indicate standard error of the mean, *SSDD* short-sleep delta-deficient subtype, *NSDD* normal-sleep delta-deficient, *NNS* normal neurophysiological sleep subtype, *p < 0.05, **p < 0.01.

As the quality of physiological signals was important for investigating EEG-derived measures, we only included individuals with good quality EEG signals. Due to technical recording and EEG signal issues on the acute restriction sleep (night 2), we were only able to utilise 76 participants (SSDD: n = 21, NSDD: n = 39, NNS: n = 16) from the 84 insomnia participant recordings that had adequate EEG signal quality for measurement of EEG power. MANCOVA analyses showed the global delta power, as well as delta power in the left and right hemispheres, increased significantly during NREM during the acute sleep restricted night in SSDD and NSDD relative to the habitual sleep opportunity (night 1), but no effects were observed for participants with NNS (Fig. 5). In each subtype, there was no significant correlation between delta power and TST, WASO, or SOL. We also found SSDD increased theta on the second night (27.7 vs. 31.5 uV^2^/Hz on night 1 and 2, respectively, *p* < 0.05). Delta power during each sleep stage on the bedtime restriction night are shown in Supplementary Table 5. There were no differences in the IAI between the nights across all insomnia subtypes (Supplementary Figure 2).

**Figure 6:**
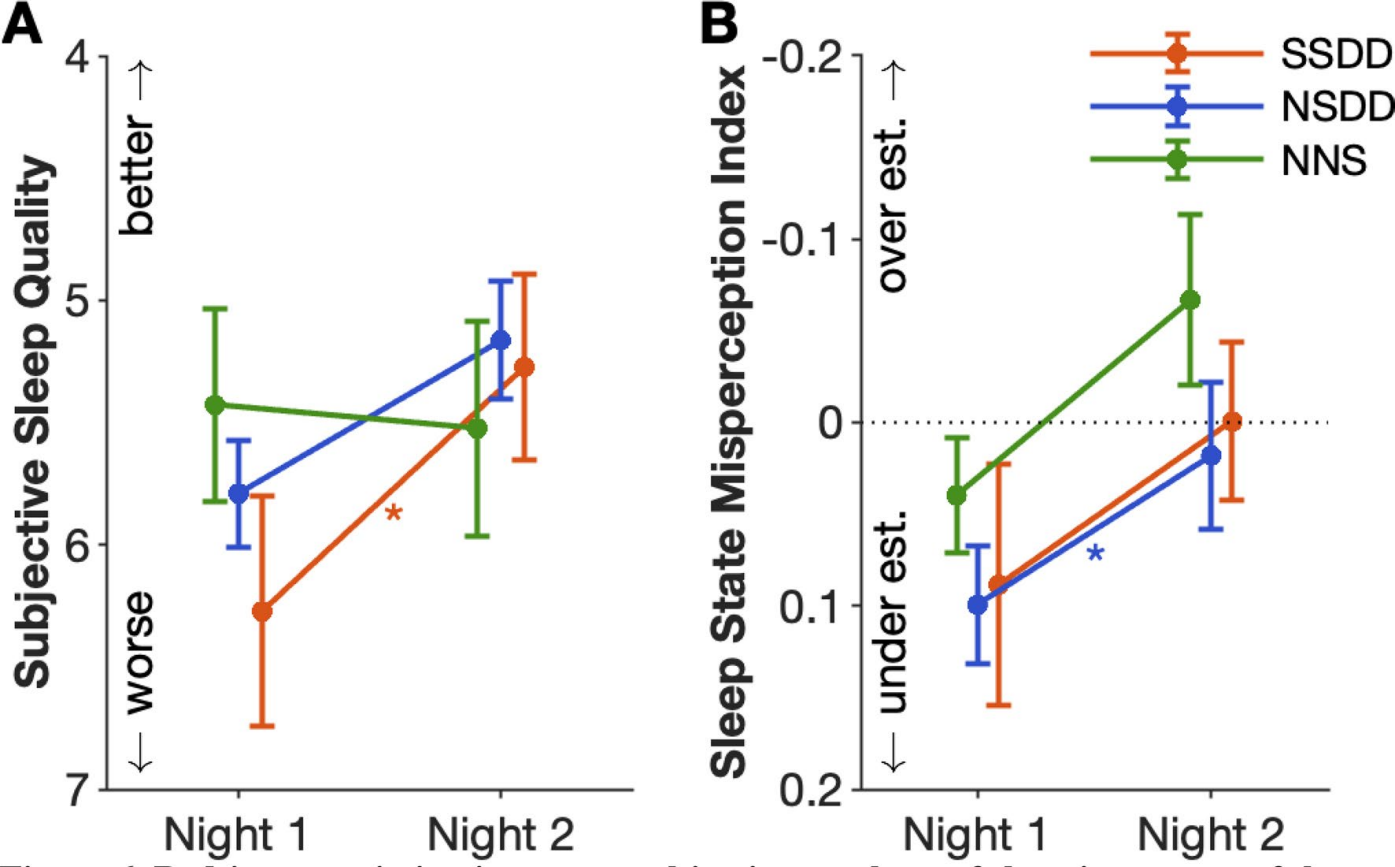
Bedtime restriction improves subjective markers of sleep in two out of three insomnia subtypes. Subjective sleep quality rating (**A**) and sleep state misperception (**B**) between habitual sleep (night 1) and acute sleep restriction (night 2) for the three insomnia subtypes. Error bars indicate standard error of the mean, *SSDD* short-sleep delta-deficient subtype, *NSDD* normal-sleep delta-deficient, *NNS* normal neurophysiological sleep subtype, * *p* < 0.05.

The improvements in sleep macro-architecture and EEG spectral-power measures during the acute sleep restriction night were associated with improvements in subjective sleep quality for SSDD (p = 0.03), with a trend observed for NSDD (Fig. 6A, p = 0.057). NSDD also showed reduced sleep-state misperception between the different sleep studies (Fig. 6B; *p* = 0.03). NNS did not report any changes in sleep quality or sleep misperception between the sleep studies. There was no significant correlation between SSM and TST, WASO, or SOL in each subtype. Supplementary Table 6 summarises the effect of acute sleep restriction on the three insomnia subtypes.

## Discussion

This exploratory analysis identified three novel insomnia subtypes based on PSG-derived sleep macroarchitecture and EEG spectral power variables. Overall, all insomnia subtypes had higher ISI, PSQI, DASS, and FSS scores than healthy controls, which is consistent with previous research^45^. These subtypes have distinct objective sleep duration, coupled with differences in delta power during NREM and REM sleep and importantly differences in subjective sleep. Our findings were unaffected by age or gender. Only the SSDD subtype showed aberrant sleep macroarchitecture along with reductions in EEG-delta power during NREM and REM sleep. The NSDD subtype also showed this reduced EEG-delta power, but this did not result in aberrant sleep macroarchitecture. Furthermore, using acute sleep restriction to reduce sleep opportunity, both SSDD and NSDD subtypes showed improvements in SE, SOL, WASO, delta power and subjective sleep ratings. Finally, the NNS subtype seemed to have “normal” sleep with no apparent differences in sleep macrostructure or EEG-power as compared to controls and showed no changes in delta power with sleep restriction. Given the absence of abnormal neurophysiological findings in NNS, we speculate that this subtype will only show neurophysiological abnormalities using higher granularity EEG measures, such as high-density EEG (HdEEG) studies to explain their insomnia symptomology^30^. Collectively, our exploratory findings are the first to show insomnia subtypes that have distinctive patterns in sleep duration, coupled with differences in delta power and subjective reports (Supplementary Tables 3 and 6). These findings add to the potential use of EEG phenotyping in personalised management of insomnia.

### Insomnia subtypes

Our data-driven approach identified at least one insomnia subtype with short (< 6 h, SSDD) and two with normal sleep duration (> 6 h, NSDD and NNS). The SSDD in our study aligns to some extent with the proposed short-sleep insomnia phenotype based on objective sleep duration^54^. This phenotype was originally proposed to explain a biologically severe phenotype (< 6 h TST) with significant hyperarousal and increased morbidity risk. Subsequently, there have been a number of studies that have shown increased cardiometabolic risk^3,22^, attenuated cognitive behavioural therapy response^53^, and brain metabolism differences^36^ between the short-, and normal-sleep duration insomnia.

Both insomnia SSDD and NSDD subtypes significantly underestimated sleep duration compared with controls during a habitual sleep night. They also showed greater sleep misperception coupled with reduced delta power in NREM sleep compared to NNS and controls. Some have proposed that sleep‐state misperception may represent one insomnia subtype^33^, but our observations suggest that both SSDD and NSDD report misperception. However, recent evidence shows that individuals with insomnia who underestimate sleep duration may actually be accurately reporting sleep disturbance^30^. They used HdEEG in 10 participants and found that sleep was associated with a shift from low to high frequency spectral power in central and posterior brain regions indicative of wake-like activity. Our findings support these results and suggest that sleep misperception, based on sleep duration, may need to be reconsidered as sleep mismeasurement, especially in relation to subtype differences in insomnia. Although NNS reported insomnia (Insomnia Severity Index (ISI) and Pittsburgh Sleep Quality Index (PSQI)), there was no clear evidence of SSM and similar PSG and EEG spectral power findings as controls. It is possible that this subtype has a lesser degree of abnormality on HdEEG testing but this is entirely speculative and will require larger studies of insomnia subtypes.

### Acute sleep restriction

With the acute sleep restriction, we found insomnia SSDD and NSDD not only increased delta power but also showed improvements in several clinical outcomes, such as objective sleep efficiency, subjective sleep quality and sleep misperception. Despite some participants not completing the acute sleep restriction night, the characteristics of each subtype did not differ from the original dataset on first night in terms of demographics, clinical characteristics, sleep architecture, or EEG delta power variables.

Our findings show that one night of acute sleep restriction can significantly increase SWA in delta-deficient subtypes and improve sleep quality. This suggests that sleep restriction used in clinical practice to treat insomnia can increased SWA and subjective sleep quality in individuals with insomnia, especially those with deficits in SWA as shown in the SSDD and NSDD subtypes. These findings align with research showing that sleep restriction therapy improves both objective and subjective sleep in insomnia^13,15,37^. Whilst night-to-night variability in insomnia may influence results, most participants responded to acute sleep restriction with improvements in subjective sleep perception (Supplementary Figure 3). Our results support the hypothesis that the impaired homeostatic sleep function in insomnia may be corrected using behavioural treatment, such as sleep restriction, to improve sleep quantity and quality^8^. However, further research with long-term sleep restriction interventions is needed to elucidate the neurophysiological processes in insomnia^34^.

### Interhemispheric asymmetry

Interhemispheric asymmetry did not contribute meaningfully to identifying insomnia subtypes. Previous work^52^ found reduced SWA in the left hemisphere compared to the right hemisphere in healthy young participants experiencing their first night in a sleep laboratory (first night effect). This suggests that interhemispheric asymmetry maybe more related to immediate disturbed sleep in young healthy controls rather than to chronic sleep disturbances. It is possible we did not observe interhemispheric asymmetry as we used the whole hemisphere which may not adequately identify regional asymmetry between subtypes. Whilst we did not find associations between interhemispheric asymmetry and subjective sleep quality, further research is needed to clarify the role of interhemispheric asymmetry in insomnia and by exploring regional EEG brain activity using HdEEG^10^.

### Limitations

There are several limitations in this study. This was an exploratory study using a clustering approach to differentiate insomnia subtypes was based on a single night PSG. Spectral EEG activity may have been affected by first night effect or influenced by reverse first-night effect in insomnia. The reliability of neurophysiological subtypes in insomnia would need to be explored in future studies by using multiple sleep nights. Our data are from cross-sectional analyses and the stability of the subtypes needs to be assessed by using longitudinal designs. We observed three subtype clusters from 99 insomnia patients, however, a larger dataset of participants and controls will be required to confirm the subtypes and clinical significance with response to various types of therapies and may show differences in subjective sleep quality and sleep misperception. There may have been an order effect based on participants completing a habitual sleep night followed by a consecutive sleep restricted night. Although others have examined sleep restriction and found similar results^8^, this needs to be tested in larger samples and with matched healthy sleeping controls.

### Implications for treatment and conclusion

It remains to be seen whether this or other ID subtyping can assist in targeting specific insomnia therapies to patients. The improvements in both sleep, spectral power, and subjective reports suggest that the behavioural therapy of sleep restriction could be universally applied to all insomnia patients but would significantly improve sleep architecture and sleep perception in those insomnia subtype patients with delta power deficits. Furthermore, SSDD may require a shorter period of hypnotic medication to improve the impaired homeostatic sleep drive, and slow wave enhancement therapy for both SSDD and NSDD based on the spectral power deficits. Clinical trials are needed to test these hypotheses.

In conclusion, our data-driven classifications of objective PSG sleep duration and EEG spectral power revealed three neurophysiological insomnia subtypes, highlighting potential neural mechanisms underlying sleep misperception in insomnia disorder. The results suggest that SWA may be deficient in some subtypes and can be related to the subjective complaints reported in insomnia. In addition to the two subtypes with deficits in delta power during NREM sleep, we observed a third insomnia subtype with normal PSG and EEG spectral power patterns. This will provide insights into the biological mechanisms underpinning insomnia presentations, especially related to misperception, a central tenet of many insomnia patients.

## Methods

### Participants

Insomnia participants were recruited from the community and sleep clinics at the Woolcock Institute of Medical Research, Sydney and Adelaide Institute of Sleep Health and Flinders University, Adelaide, Australia**.** They were initially screened online for eligibility on the basis of an Insomnia Severity Index (ISI) score ≥ 10. Eligible participants were then invited to attend the sleep clinic for a comprehensive sleep assessment by a sleep physician or sleep psychologist who diagnosed Insomnia Disorder using DSM-5^1^, with participants having difficulty initiating or maintaining sleep or waking up too early for at least three nights per week and greater than three months, with adequate opportunity for sleep coupled with daytime impairment related to the sleep difficulty. Participants also needed to be over 18 years of age, fluent in English, and have a habitual bedtime before midnight determined by a 7-day assessment with sleep diaries and actigraphy (Actiwatch-2, Actiwatch Spectrum; Respironics, Bend OR USA Spectrum and AW 64). Participants were excluded if they were being actively treated for sleep disorders, self-reported illicit substance usage, reported excessive consumption of alcohol or caffeine, had major or unstable psychiatric disorders or cognitive impairment as evaluated by the sleep physician/psychologist, were shift-workers, had transmeridian travel in the past 2 months (two or more time zones traversed) or were pregnant or lactating. Control participants were recruited from the community using online research advertisements and completed the online screening for an ISI < 10. They were then telephone screened for study inclusion and exclusion criteria. This was a sub-study from a larger study which was prospectively registered on the Australian New Zealand Clinical Trial Registry (ANZCTR 12612000049875). Ethical approval was provided by the Royal Prince Alfred Hospital Ethics Review Committee, Sydney, Australia (Protocol No X11-0392 & HREC/11/RPAH/620). Participants provided written informed consent prior to study commencement. All methods were followed in accordance with approved rules and regulations.

### Experimental protocol

Insomnia participants attended the sleep laboratory for two consecutive nights with full attended overnight PSG on night one. Participants were given the opportunity to sleep according to habitual bed- and rise times calculated from a prior 7-day sleep diary. All participants completed baseline questionnaires including the Insomnia Severity Index (ISI,^2^, Pittsburgh Sleep Quality Index (PSQI,^6^, Depression, Anxiety and Stress Scale (DASS,^18^, Epworth Sleepiness Scale (ESS,^21^, and Fatigue Severity Scale (FSS,^28^. Participants left the laboratory in the morning and returned in the early evening for a second overnight EEG sleep study commencing 2-h past habitual bedtime (acute sleep restriction). Participants were woken at their habitual rise time. During the acute sleep restriction night, we used a similar protocol as previous studies to delay bedtime and retain normal wake time^55^. To compare insomnia neurophysiology, we recruited healthy adults as controls for the first night overnight sleep.

### Measurements

#### Polysomnography

The PSG montage was identical on the two nights, and consisted of EEG electrodes placed at F3, Fz, F4, C3, C4, Pz, O1, Oz, O2 and mastoid channels at M1 and M2. PSG also included electromyographic (submental) and electrooculographic (horizontal and vertical) electrodes, electrocardiogram (ECG), nasal pressure, and finger pulse oximetry. PSGs were acquired using Embla (Mortara, Broomfield, CO, USA) or Compumedics (Grael 4 K/Somte PSG, Charlotte, NC, USA) systems and EEG signals were sampled at 512 Hz. Sleep studies were scored and staged in 30-s epochs by an experienced sleep technologist using the American Academy of Sleep Medicine criteria^20^.

#### EEG spectral power analysis

**Figure 7:**
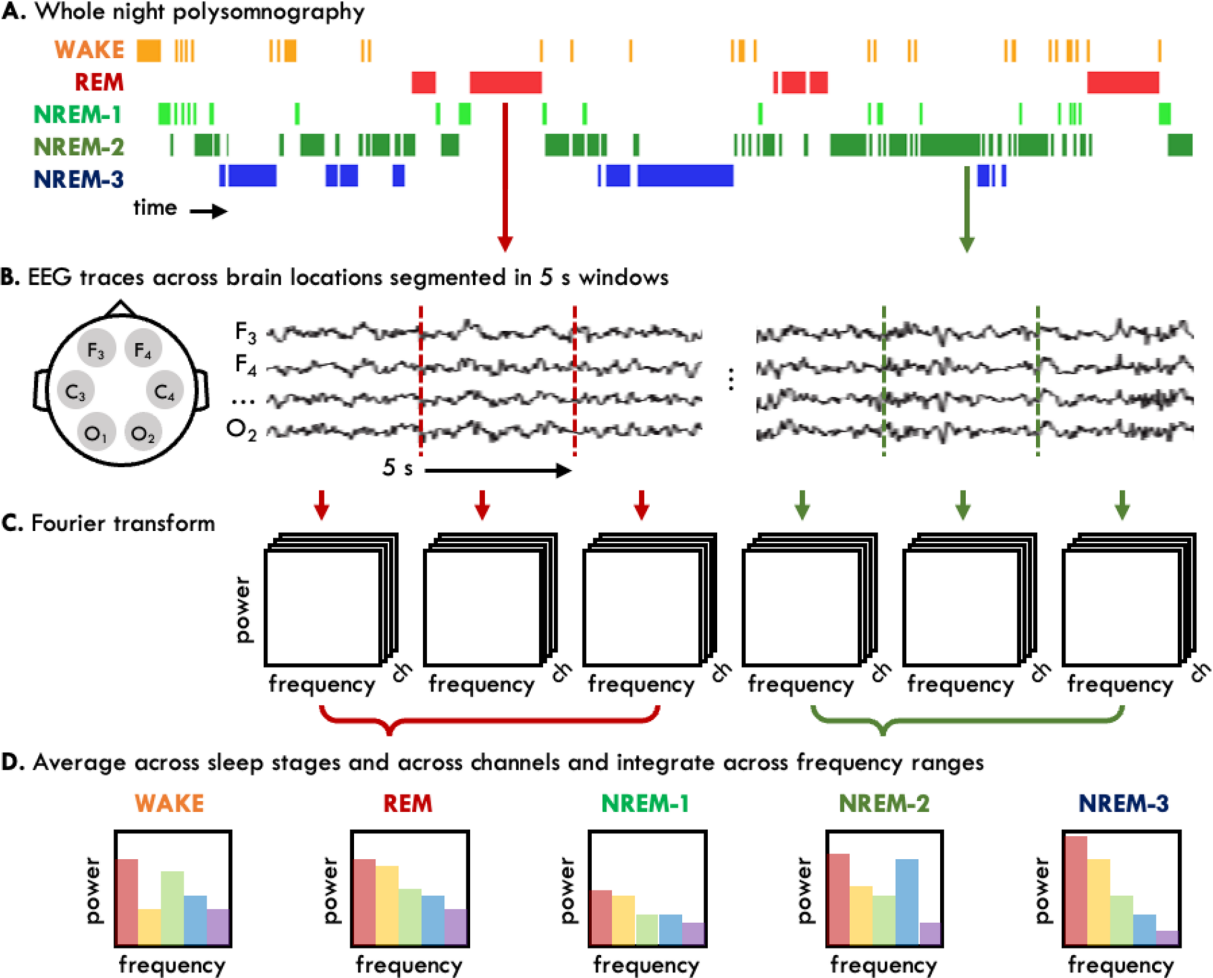
EEG data analysis pipeline. (**A**) Whole-night polysomnography recordings were staged into wake, REM and NREM-1, -2 and -3 sleep stages. (**B**) EEG traces from 6 channel locations were segmented into 5-s epochs. Epochs containing artefact were removed. (**C**) Each 5-s epoch was subjected to fast Fourier transform to obtain power spectra for each EEG channel. (**D**) Power spectra were averaged across sleep stages and integrated across standard frequency bands (coloured bars). Subsequently, the absolute power at each frequency band was averaged across all channels to obtain global EEG power, and across left and right channels to obtain intra-hemispheric EEG power. *s* seconds, *Ch* channel.

PSGs were exported to European Data Format along with synchronised sleep staging files. All-night EEG recordings were processed using a validated automated artefact detection algorithm to identify EEG artefact on consecutive non-overlapping 5 s epochs^11^. We utilised advanced algorithms to evaluate the quality of each participant's sleep EEG signal, and visually-inspected EEG data quality. Verification was conducted by a trained researcher, and single EEG traces were excluded if they were of poor quality for more than 25% of the recording. The artefact-free EEG traces were segmented into 5-s epochs, which were transformed to power spectra using a standard fast Fourier transform. The power spectra were then averaged for each sleep stage. Absolute EEG spectral power (μV^2^) was calculated across five frequency ranges: delta (0.5–4.5 Hz), theta (4.5–8 Hz), alpha (8–12 Hz), sigma (12–15 Hz), and beta (15–32 Hz). Global EEG power was calculated by averaging data from up to 6 channels (F3-M2, F4-M1, C3-M2, C4-M1, O1-M2, O2-M1). EEG power in the left (average of F3-M2, C3-M2, O1-M2) and right (F4-M1, C4-M1, O2-M1) hemispheres was also calculated for all frequency ranges (Fig. 7).

#### Interhemispheric asymmetry index

The interhemispheric asymmetry index (IAI) was calculated for the delta frequency range (0.5–4.5 Hz) according to a previously described method^52^. The index was calculated as the ratio of delta power difference between the left (F3-M2, C3-M2, O1-M2) and right hemispheres (F4-M1, C4-M1, O2-M1) to the average delta power within each sleep stage and wakefulness:

$$IAI_{s} = \frac{{\mathop \sum \nolimits_{i = 1}^{n} x_{i} - \mathop \sum \nolimits_{j = 1}^{m} x_{j} }}{{\mathop \sum \nolimits_{i = 1}^{n} x_{i} + \mathop \sum \nolimits_{j = 1}^{m} x_{j} }}$$

where $x$ is the average delta power in sleep stage *s*, and *n* and *m* is the channel number in the left and right hemisphere, respectively. A positive IAI indicated greater delta activity in left hemisphere compared the right and vice versa.

#### Subjective measurements

All participants were asked to self-report their subjective SOL, WASO, TST and a sleep quality rating (SQ, from 1 ‘best sleep ever’ to 9 ‘worst sleep ever’) for the two laboratory nights. For each participant ($s$), sleep-state misperception (SSM) was calculated as follows:

$$SSM_{s} { = }\frac{{\user2{objective\,TST}_{{\varvec{s}}} - \user2{subjective\,TST}_{{\varvec{s}}} }}{{\user2{objective\,TST}_{{\varvec{s}}} }}$$

A positive SSM value indicated an underestimation of TST whereas a negative value indicated an overestimation of TST.

### Data analysis

#### Insomnia subtype definition using clustering techniques

One of the main issues with insomnia populations is the heterogeneity in objective and subjective sleep measures. To address this problem, we used cluster analysis to form homogeneous insomnia subtypes based on PSG and qEEG variables from the insomnia group (n = 99) on the habit sleep night at laboratory. To identify these clusters, we used thirty-three variables in total, which included standard sleep macrostructure variables, time spend in each sleep stage, global absolute delta power, left and right hemispheric delta power, and the IAI (Supplementary Table 7). To reduce the collinearity across variables and number of features used for cluster analysis while retaining the majority of variance, we normalized the variables by converting raw scores to *z*-scores and applied principal component analysis (PCA,^23^. PCA is a classic variable reduction method where a large number of variables are linearly combined into a few components that still represent the majority of original variance. It has been used extensively to quantify phenotypes of complex diseases and identify biomarkers of disease risk based on the genetic information^46^ or clinic symptoms^38^. By reducing a large number of variables into a fewer principal components (PCs), we retained the majority of the variance in the dataset without losing the meaningful information, which simplifies downstream cluster analysis of participants. Supplementary Table 8 showed the eigenvalues and individual explained variance of Top 10 PCs.

Similar to our previous research^36^, we used a hierarchical agglomerative clustering method—with Ward’s minimum variance method using Euclidean distance—to determine the cluster dendrogram of insomnia subtypes. The agglomerative clustering procedure maps the principal components in an *M*-dimensional space, where *M* is the number of principal components, and starts by assuming that each participant belongs to its own cluster i.e., 50 participants = 50 clusters. Next, the algorithm computes the Euclidean distance between each pair of clusters (participants). Ward’s clustering method considers all possible combinations of clusters and minimises the variance within each cluster, whilst maximising the Euclidean distances between clusters^56^. Finally, we used permutation tests to determine the optimal number of clusters^16^. To do so, we randomly permuted (scrambled) the values for each principal component across all participants and performed the same cluster analysis. The permutation procedure was repeated 10,000 times. For each increasing cutting level of the dendrogram, we calculated the probability of observing its number of clusters based on the histogram of random permutations. The first cutting level where the probability was smaller than 0.05 was taken as the optimal number of clusters. The cluster analysis was performed using R (version 3.6.1) with the *hclust* package^32^.

#### Statistical analysis

We compared the demographic characteristics of the insomnia subtypes and controls using Chi-square or Fisher’s exact test for categorical variables and *t*-tests or Wilcoxon signed-rank test for continuous variables. Multivariate analysis model was used in the current study. The demographics, clinic characteristics of insomnia and controls were analyzed by a mixed-effect MANOVA using the maximum likelihood estimation. The PSG and qEEG characteristics of the insomnia subtypes were compared to the controls with MANCOVA analysis. A mixed-effect MANCOVA with three independent variables (three insomnia subtypes) and two repeated measure variables (night one and two) was used to examine the bedtime restriction effect in PSG and qEEG characteristics. We included age and gender a covariate in the MANCOVA model to adjust for the confounding variable. Turkey analysis with Bonferroni corrections was applied to post-hoc comparisons. All the statistic computations were performed using R with the *nlme* package^24^.

## Supplementary Information

Supplementary Information.
