# Supplementary material for: Insomnia subtypes characterised by objective sleep duration and NREM spectral power and the effect of acute sleep restriction: an exploratory analysis
Source: Sci Rep. 2021 Dec 21;11:24331. doi: 10.1038/s41598-021-03564-6 (PMC8692344; doi:10.1038/s41598-021-03564-6)
Supplement: Supplementary file 1 — Supplementary Information. [file 41598_2021_3564_MOESM1_ESM.docx]

**Table S1.** Polysomnographic sleep architecture variables on the habitual sleep study for insomnia subtypes and controls.

| **Variables** | **Insomnia** | | | **Control**  **n=25** |
| --- | --- | --- | --- | --- |
|  | **SSDD**  **(n=26)** | **NSDD**  **(n=51)** | **NNS**  **(n=22)** |  |
| NREM (%) | 82.4±0.9 | 75.6±0.9 | 81.9±1.2 | 78.3±1.6 |
| REM (%) | 17.6±0.9 | 24.4±0.9 | 18.1±1.2 | 21.7±1.6 |
| NREM (min) | 246.3±10.3 | 280.2±5.6 | 314.9±11.4 | 277.3±12.1 |
| REM (min) | 52.9±3.8^*^ | 91.2±3.9 | 71.0±5.9 | 77.7±5.8 |
| Arousal index (TST) | 25.4±2.4^*^ | 13.5±0.8 | 15.1±1.7 | 12.1±1.4 |
| Arousal Index (NREM) | 26.5±2.8^*^ | 12.7±0.8 | 15.3±1.9 | 11.2±1.7 |
| Arousal Index (REM) | 20.2±2.1 | 15.7±1.4 | 15.5±2.6 | 16.2±1.7 |

Note: Values are expressed as mean ± S.E.M., SSDD - short-sleep delta-deficient subtype, NSDD - normal-sleep delta-deficient, NNS - normal neurophysiological sleep subtype, ^*^ *p*<0.05 denotes the significant difference compared to controls.

**Table S2.** Spectral EEG delta power during the habitual sleep study for insomnia subtypes and controls.

| **Spectral power (uV^2^)** | **Insomnia** | | | **Control**  **n=25** |
| --- | --- | --- | --- | --- |
|  | **SSDD**  **(n=26)** | **NSDD**  **(n=51)** | **NNS**  **(n=22)** |  |
| RH Wake Stage | 100.1±11.2^*^ | 84.7±6.3^*^ | 241.5±32.0 | 267.7±93.2 |
| LH Wake Stage | 92.7±9.7^*^ | 84.3±6.1^*^ | 201.0±31.4 | 229.4±85.3 |
| RH Stage 1 | 68.2±6.6^*^ | 59.2±3.8^*^ | 150.5±27.1 | 139.2±28.7 |
| LH Stage 1 | 66.3±7.1 | 60.6±4.8^*^ | 128.7±26.2 | 108.9±12.5 |
| RH NREM | 231±21.2^*^ | 240±15.6^*^ | 358±47.7 | 396±65 |
| LH NREM | 239±24.5^*^ | 240±15.2^*^ | 338±43.6 | 359±50.9 |
| RH REM | 63.4±4.3^*^ | 53.2±2.7^*^ | 94.36±10.0 | 109.2±20.9 |
| LH REM | 55.7±3.3^*^ | 53.5±3.6^*^ | 82.4±8.4 | 117.4±29.5 |

Note. Values are expressed as mean ± S.E.M., SSDD - short-sleep delta-deficient subtype, NSDD - normal-sleep delta-deficient, NNS - normal neurophysiological sleep subtype, ^*^ *p*<0.05 denotes the significant difference compared to controls. Abbreviations: RH = right hemisphere, LH = left hemisphere.

**Table S3.** Polysomnographic and EEG spectral power variables for the three insomnia subtypes.

| Variable | SSDD | NSDD | NNS |
| --- | --- | --- | --- |
| Characteristics | short sleep/  delta power deficit | normal sleep/  delta power deficit | normal sleep/ no delta power deficit |
| SE | ↓ | ⎯ | ⎯ |
| TST | ↓ | ⎯ | ⎯ |
| WASO | ↑ | ⎯ | ⎯ |
| SOL | ⎯ | ⎯ | ⎯ |
| NREM EEG delta power | ↓ | ↓ | ⎯ |
| NREM EEG sigma power | ⎯ | ⎯ | ⎯ |
| NREM EEG theta power | ⎯ | ⎯ | ⎯ |
| Subjective sleep quality | ⎯ | ⎯ | ⎯ |
| Sleep misperception index (underestimate) | ↑ | ↑ | ⎯ |

Note. The arrow indicates a statistically significant increase “↑” or decrease “↓” compare to the controls. The symbol “⎯” indicates the no difference between the subtype and controls. SE – sleep efficiency; TST – total sleep time; WASO – wake after sleep onset; SOL – sleep onset latency. SSDD - short-sleep delta-deficient subtype, NSDD - normal-sleep delta-deficient, NNS - normal neurophysiological sleep subtype.

**Table S4.** Polysomnographic sleep architecture variables in the three insomnia subtypes during acute sleep restriction.

| **Variable** | **Insomnia** | | |
| --- | --- | --- | --- |
|  | **SSDD**  **(n=21)** | **NSDD**  **(n=42)** | **NNS**  **(n=21)** |
| TST (min) | 276.4±10.3 | 259.2±10.9*** | 284.7±10.7*** |
| SOL (min) | 7.6±0.9*** | 10.6±2.4 | 14.5±2.5 |
| WASO (min) | 43.3±5*** | 31.6±4.8*** | 24.8±4.25** |
| SE (%) | 86±1.4*** | 88.5±1.6* | 90.3±1.3* |
| Total arousal index in TST | 13.9±1.8*** | 9.5±0.6** | 9.2±0.8** |
| Total arousal count in TST | 64.9±8.5*** | 42.3±3*** | 45.5±4.4*** |
| NREM (%) | 78.8±1.1* | 72.7±1.2* | 78.7±1.2* |
| REM (%) | 21.2±1.1* | 27.3±1.2* | 21.3±1.2* |
| NREM (min) | 217±8.3* | 187±6*** | 223±7.3*** |
| REM (min) | 58.9±4.1 | 72.2±4.5** | 62.1±4.8 |
| Stage 1 % | 5.3±0.8*** | 3.7±0.5** | 3.4±0.5 |
| Stage 1 (min) | 14.5±2.4*** | 9.1±0.9*** | 9.8±1.6** |
| Stage 2 % | 39.7±2** | 32.3±1.2*** | 40.9±1.9*** |
| Stage 2 (min) | 109±6.3** | 84.5±4.6*** | 117 ±7.2*** |
| Stage 3 % | 33.8±2.3** | 36.8±1.2*** | 34.4±2.3*** |
| Stage 3 (min) | 94.1±7.7* | 93.4±3.3** | 96.2±6.5 |
| Total Arousals Index in NREM | 13.6±2.1*** | 8.5±0.6** | 8.4±0.8*** |
| Total Arousals Index in REM | 14.9±2.2* | 11.2±1.1** | 12.2±1.4 |

Values are expressed as mean ± S.E.M.. * *p* < 0.05, ** *p* < 0.01, *** *p* < 0.001 denote the significant difference between two nights after Bonferroni correction. TST – total sleep time; SOL – sleep onset latency; WASO – wake after sleep onset; SOL – sleep onset latency; SE – sleep efficiency. SSDD - short-sleep delta-deficient subtype, NSDD - normal-sleep delta-deficient, NNS - normal neurophysiological sleep subtype.

**Table S5.** EEG spectral delta power between the insomnia subtypes during acute sleep restriction.

| **Spectral Power(uV^2^)** | **Insomnia** | | |
| --- | --- | --- | --- |
|  | **SSDD**  **(n=21)** | **NSDD**  **(n=39)** | **NNS**  **(n=16)** |
| LH Wake Stage | 161.8±43.1 | 122.2±15.2 | 170±31 |
| RH Wake Stage | 165.3±48.4 | 108.9±10.3 | 109.4±22.6 |
| LH Stage 1 | 91.5±12.8 | 73.22±7 | 112.5±23 |
| RH Stage 1 | 84.1±10.5 | 68.46±5.7 | 109.4±22.6 |
| LH NREM | 281.5±35.4^*^ | 287.6±24.7^*^ | 329.5±48.4 |
| RH NREM | 281.6±7.7^*^ | 285.3±5.4^*^ | 337.6±9.7 |
| LH REM | 67.2±31.1 | 56.5±24.7 | 70.8±48.4 |
| RH REM | 68.8±6.8 | 52.8±3.2 | 66.1±6.7^*^ |

Values are expressed as mean ± S.E.M.. ^*^ *p* < 0.05 denotes the significant difference between night 1 and night 2 after Bonferroni correction. Abbreviations: RH = right hemisphere, LH = left hemisphere. SSDD - short-sleep delta-deficient subtype, NSDD - normal-sleep delta-deficient, NNS - normal neurophysiological sleep subtype.

**Table S6.** The effect of acute sleep restriction (2-hours reduced bedtime) on the three insomnia subtypes’ polysomnographic sleep architecture and EEG power spectral variables.

| Variable | SSDD | NSDD | NNS |
| --- | --- | --- | --- |
| Characteristics | short sleep/  delta power deficit | normal sleep/  delta power deficit | normal sleep/ no delta power deficit |
| SE | ↑ | ↑ | ↑ |
| WASO | ↓ | ↓ | ↓ |
| SOL | ↓ | ⎯ | ⎯ |
| NREM EEG delta power | ↑ | ↑ | ⎯ |
| NREM EEG theta power | ↑ | ⎯ | ⎯ |
| NREM EEG sigma power | ⎯ | ⎯ | ⎯ |
| Subjective sleep quality | ↑ | ⎯ | ⎯ |
| Sleep misperception index (underestimate) | ⎯ | ↓ | ⎯ |

Note. The arrow indicates a statistically significant increase “↑” or decrease “↓” compare to the habitual sleep study (night 1). The symbol “⎯” indicates the no difference between habitual sleep study (night 1) and acute sleep restriction (night 2). SE – sleep efficiency; WASO – wake after sleep onset; SOL – sleep onset latency. SSDD - short-sleep delta-deficient subtype, NSDD - normal-sleep delta-deficient, NNS - normal neurophysiological sleep subtype.

**Table S7.** Sleep architecture variables of insomnia for clustering from the habitual sleep study data.

| **Major types** | **Selected variables** |
| --- | --- |
| Polysomnography | Delta in the wake, S1, NREM, and REM stages in the left hemisphere  Delta in the wake, S1, NREM, and REM stages in the right hemisphere |
| Interhemispheric asymmetry index (IAI) | IAI in the wake, S1, NREM, and REM stages |
| Sleep architecture disturbance | Sleep latency (min)  Sleep efficiency (%)  Stage 1 %', 'Stage 1 (min)  Stage 2 %', 'Stage 2 (min)  Stage 3 %’, 'Stage 3 (min)  NREM %', 'NREM (min)  REM %', 'REM (min)  REM Latency (min)  Time in Bed (min)  Total Sleep Time (min)  Total Time Awake During Sleep Period (min)  REM Latency (min) |
| Autonomic dysfunction | Total Arousal Count in TST  Total Arousals Index in NREM  Total Arousals Index in REM  Total Arousals Index in TST |

**Table S8.** The egenvalues and explained variance of Top 10 of principal components analysis.

|  | PC1 | PC2 | PC3 | PC4 | PC5 | PC6 | PC7 | PC8 | PC9 | PC10 |
| --- | --- | --- | --- | --- | --- | --- | --- | --- | --- | --- |
| Eigenvalue | 6 | 5.2 | 4.8 | 3.2 | 2.4 | 2 | 1.7 | 1.2 | 1.1 | 1 |
| Explained variance (%) | 18.7 | 16.1 | 15 | 9.8 | 7.4 | 6.4 | 5.3 | 3.8 | 3.1 | 2.6 |


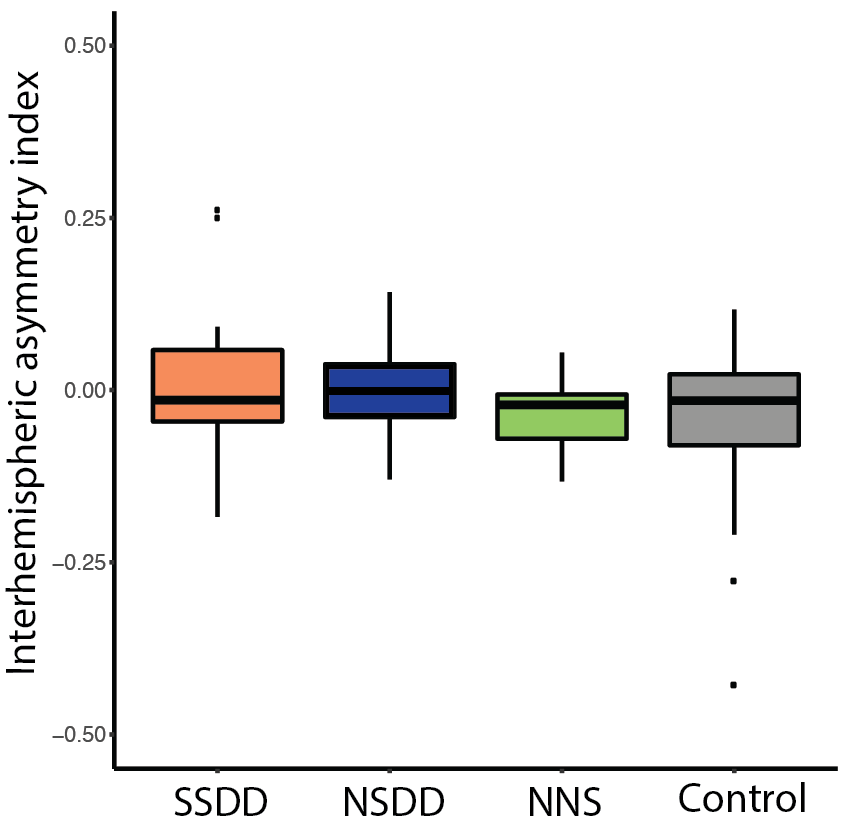


**Figure S1.** Interhemispheric asymmetry index for insomnia subtypes and controls across the habitual sleep night. Interhemispheric asymmetry index was calculated as [left delta power - right delta power]/[left delta power + right delta power]. The upper and lower whiskers indicate the value with Q3 + 1.5 x IQR and the value with Q1 - 1.5 x IQR, respectively. Black dots indicate outliers. SSDD - short-sleep delta-deficient subtype, NSDD - normal-sleep delta-deficient, NNS - normal neurophysiological sleep subtype.

**
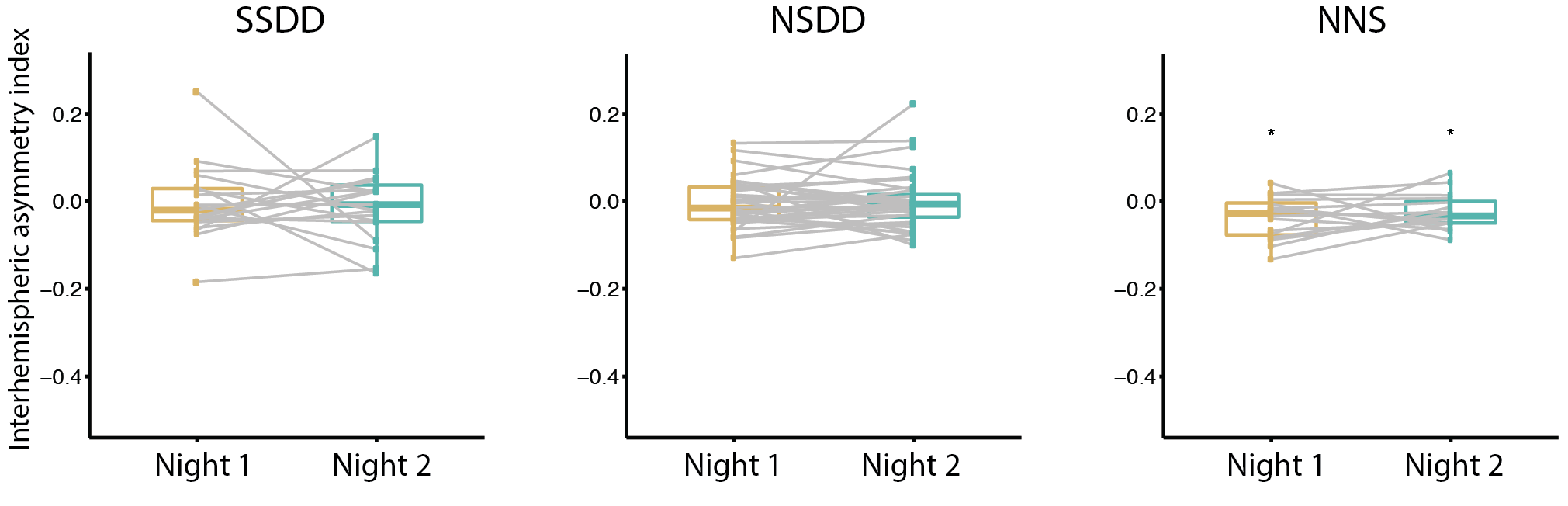
**

**Figure S2.** Interhemispheric asymmetry index for insomnia subtypes between habitual sleep (night 1) and acute sleep restriction (night 2). Box plot denotes the median, first quartile and third quartile of the IAI on night one and two. Each dot denotes one participant. The gray line indicates the change of IAI value for a participant between two nights. SSDD - short-sleep delta-deficient subtype, NSDD - normal-sleep delta-deficient, NNS - normal neurophysiological sleep subtype, * denotes the significant difference of average IAI from 0 on night 1 and night 2 (*p*<0.05).

**
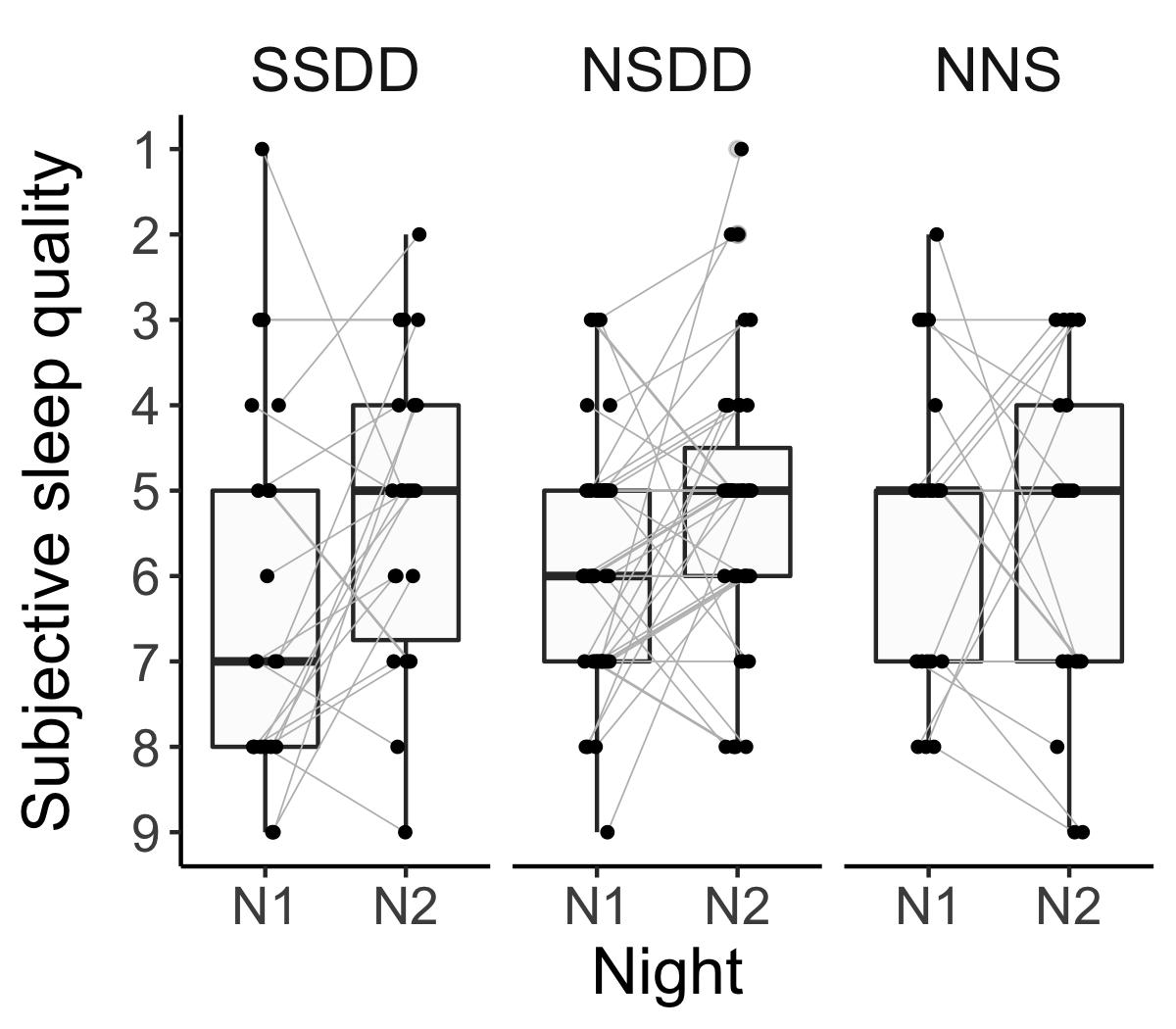
**

**Figure S3.** Subjective sleep quality (1: better; 9: worse) for insomnia subtypes between habitual sleep (night 1) and acute sleep restriction (night 2). Each dot denotes one participant. The gray line indicates the change of sleep rating for a participant between two nights. SSDD - short-sleep delta-deficient subtype, NSDD - normal-sleep delta-deficient, NNS - normal neurophysiological sleep subtype.
